# Associations between muscle-strengthening exercise and depressive and anxiety symptoms in children and adolescents

**DOI:** 10.1186/s12889-025-23663-7

**Published:** 2025-10-01

**Authors:** Lubo Zhai, Zhengyang Yang, Xingyi Yang, Sitong Chen

**Affiliations:** 1 School of Sports and Health, Shanghai Lixin University of Accounting and Finance, Shanghai, China; 2 Centre for Mental Health, Shenzhen University, Shenzhen, China

**Keywords:** Physical activity, Muscle-promoting activity, Mental illness, Depression, Anxiety

## Abstract

**Objective:**

The aim of this study was to assess the associations of days of participating in muscle-strengthening exercise (MSE) with depressive and anxiety symptoms in a large sample of Chinese children and adolescents.

**Methods:**

Cross-sectional data on 67,281 children and adolescents in Shenzhen, China was used. Days of MSE (0─7 days), depressive and anxiety symptoms, sociodemographic information, were measured using self-reported questionnaires. Multilevel multivariable logistic regressions were used to assess the associations of days of MSE with depressive and anxiety symptoms. Results were presented as odds ratio (OR) with 95% confidence interval (CI).

**Results:**

67,281 participants (13.3 ± 1.7 years, 48.1% girls) were included in this study. In the overall sample, we found a negative association of days of MSE with depressive symptoms (e.g., OR for 1 day: 0.83, 95% CI: 0.79–0.87; OR for 6 days: 0.76, 95% CI: 0.67–0.86). OR for anxiety symptoms decreased as days of MSE increased up to 6 days per week (1 day: 0.87, 95% CI: 0.83–0.92; 6 days: 0.79, 95% CI: 0.76-1.00), and a slight increase for 7 days (0.96, 95% CI: 0.89–1.05). Furthermore, the associations were mostly consistent across both sexes (i.e., boys and girls) and different school groups (i.e., primary, middle, and high school).

**Conclusion:**

Findings of this study demonstrate the negative associations of days of MSE with depressive and anxiety symptoms. It is recommended that children and adolescents can engage in more MSE for mental health promotion.

## Introduction

Depression and anxiety are the leading mental health problems in children and adolescent in the world [1–3]. Depressive symptoms, defined as persistent unhappiness or sadness, have been recognized as the most prevalent mental health problem in adolescents; anxiety, defined as excessive worry. Data from the Global Disease Burden highlights a concerning rise in depressive symptoms in adolescents compared to previous decades [4]. Similar trends were observed in anxiety in adolescents as well. According to the global data, 6.5% of children and adolescents are suffering from anxiety disorders [5]. Moreover, depressive and anxiety symptoms are related to a higher likelihood of harmful health behaviours, including obesogenic behaviour (e.g., low levels of physical activity [PA]), substance use (e.g., alcohol consumption), insufficient social engagement, and suicidality [6]. These findings underscore the urgent need for immediate measures against depressive and anxiety symptoms in children and adolescents.

There are a range of behavioral factors related to depressive and anxiety symptoms in children and adolescents [7–9]. Among these factors, PA has received growing research attention and its role in depressive and anxiety symptoms prevention and control has been studied widely [10–14]. In the literature, strong evidence demonstrates that sufficient PA is associated with lower risks for depressive and anxiety symptoms [11–13, 15, 16]. For example, Biddle et al., [10, 17] published two important review studies that synthesize the evidence on the negative association between PA and depressive symptoms. In terms of anxiety symptoms, a large body of evidence demonstrates that children and adolescents with sufficient PA are less likely to develop anxiety [10, 13, 17]; but these studies were mainly focused on aerobic or moderate to vigorous PA [14, 18].

Despite the convincing evidence regarding the association of PA with depressive and anxiety symptoms in children and adolescents, previous studies mostly focused on assessing overall or aerobic PA [10, 11, 13, 18]. This hinders researchers to understand other types of PA and their roles on mental health problem prevention and control. For this reason, researchers have called upon advocating more studies that explore the associations between different types of PA and mental health problems [19].

As a type of PA, muscle-strengthening exercise (MSE) has been an important research topic in PA epidemiology, with increasing research attention [20]. Based on the time-use epidemiological framework [21], the related research can be categorized into five domains, including (1) measurement and assessment; (2) health outcomes; (3) correlates or determinants; (4) levels and trends; (5) interventions and evaluations. From this perspective, exploring health benefits of MSE is an essential research topic [22–31]. A large number of studies have explored the associations between MSE and mental health problems in adults, especially focusing on depressive [24, 26, 27] and anxiety symptoms [28, 31]. For example, Bennie et al., [27] reported that MSE was associated with reduced prevalence ratios of mild, moderate, and moderately severe/severe depressive symptoms compared to no MSE in German adults. The other study by Bennie et al. [26], but using data from the U.S. adults, also found that having more days of MSE per week was less likely to report depressive symptoms (adjusted prevalence ratio: MSE only (range: 0.49–0.84). One randomized control trial study suggested a significant reduction in anxiety symptoms among study participants who underwent the MSE program compared to those receiving no interventions, showing the potential of MSE as an intervention for reducing anxiety in young adults [31]. However, the related studies on MSE and mental health problems in children and adolescents are limited.

The aim of this study, therefore, was to assess the associations of days of MSE with depressive and anxiety symptoms in children and adolescents using a large sample of Chinese school-aged children.

## Methods

### Study design and participants

In March 2021, the Shenzhen Education Commission carried on a comprehensive survey of students from public schools in Shenzhen, a major city in China [32, 33]. To accurately reflect the distribution of students across different districts, a multistage sampling method was employed. This study considered children and adolescents in grades 5–6 of primary school, grades 7–8 of junior middle school, and grades 10–11 of senior high school, specifically recruiting those aged 10 and above for their ability to independently complete questionnaires [34]. Ninth and twelfth graders were not included owing to their exam preparations.

Prior to the survey, both students and their guardians were briefed on its objectives and procedures. Students were assured that their participation was both voluntary and confidential, and they were instructed on how to complete the questionnaire. The survey was carried out online in a school computer lab during a class period, and took approximately 20 min. Participation was contingent on students giving electronic informed consent. Informed consent was obtained from the parent or legal guardian of any participant under 16 years of age. The study received ethical approval from the Shenzhen University Medical Ethics Committee (No. 2020005) and endorsement from the educators and administrators of the involved schools.

**Fig. 1:**
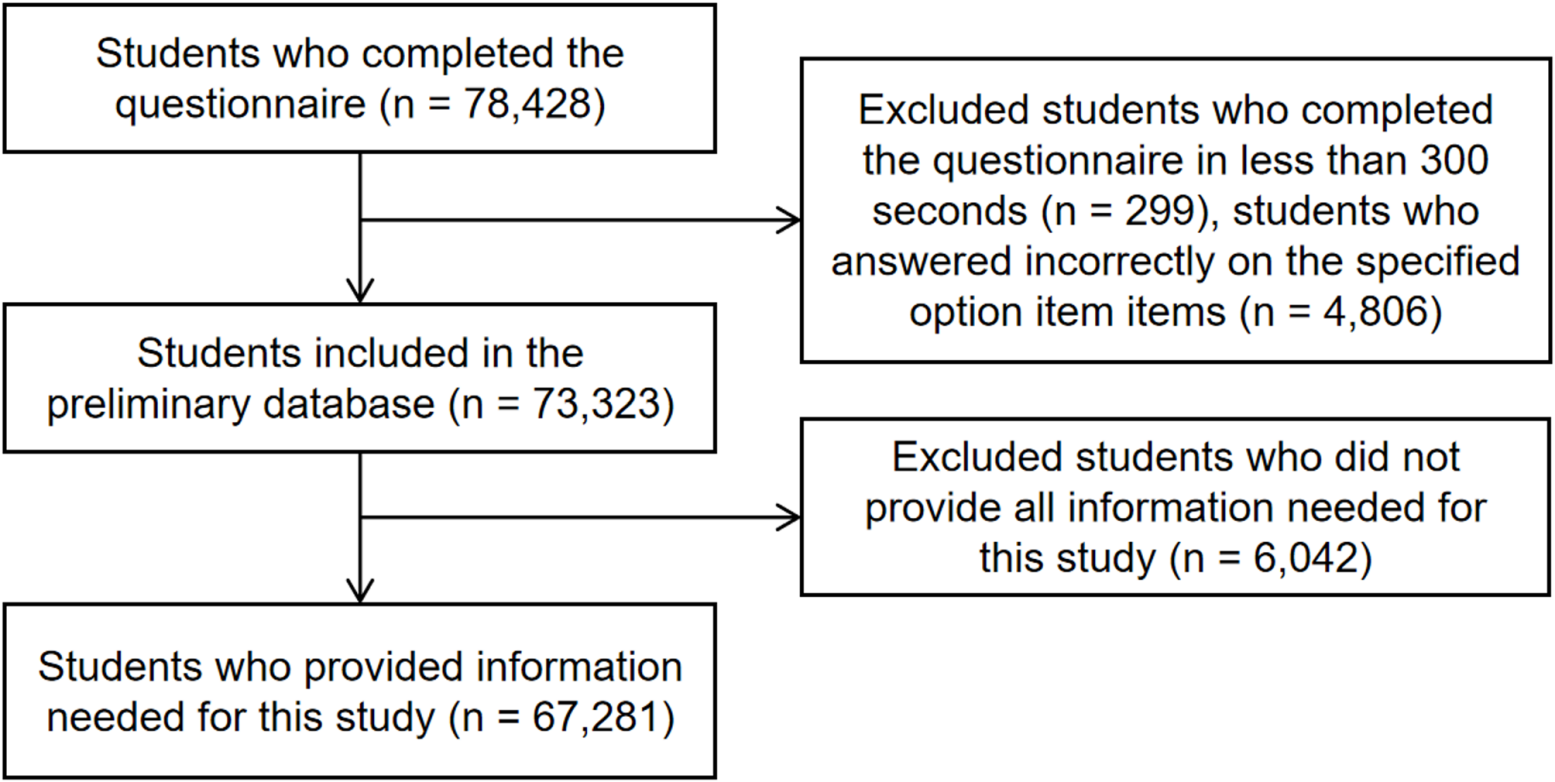
Detailed process used for cleaning invalid and missing data

In total, 79,664 children and adolescents were recruited for this study, and 78,428 submitted their questionnaires (response rate = 98.4%). After deleting invalid data from participants (e.g., did not pass the quality check items), 73,323 from 135 schools remained. According to the availability of the variables required for the current study, a total of 67,821 samples was included for analysis. Process details can be seen Fig. 1.

### Measures

#### Independent

A validated item was utilized to assess days of MSE, asking study participants, “Over the past week, on how many days did you take part in exercises designed to muscle strengthening or toning, such as push-ups, sit-ups, or weightlifting?” The response options were listed as follows: 0 signifying ‘none’, 1 for ‘1 day’, and so forth, up to 7, indicating ‘every day of the week’ [35]. This measure has been confirmed with acceptable reliability and validity in children and adolescents [36, 37].

#### Dependents

Depressive and anxiety symptoms were measured by two scales, respectively. Depressive symptoms were measured via the Chinese version of the nine-item Patient Health Questionnaire (PHQ-9). Study participants self-evaluated their symptoms in the range of 0 (not at all) to 3 (almost every day). The higher scores indicate more serious symptoms. The PHQ-9 presents good reliability and validity in the evaluation of depressive symptoms among Chinese children and adolescents [38] . Anxiety symptoms were measured using the Chinese version of the seven-item Generalized Anxiety Disorder Scale (GAD-7). The response and scoring requirements are consistent with those of the PHQ-9. The Chinese version of GAD-7 has been validated in Chinese children and adolescents [39]. Five categories of depressive symptom severity were used in the present analysis: (1) none; (2) mild; (3) moderate; and (4) moderate to severe; (5) severe. Four categories of anxiety symptom severity were used in the present analysis: (1) none; (2) mild; (3) moderate; and (4) severe [39].

#### Covariates

The following information was collected: sex (boy/girl), grade (i.e., primary school/middle school/high school), self-reported height (cm) and weight (kg), number of siblings (i.e., only one child/non-only child), family structure (both parents/single), parental education level (i.e., junior middle school or below/high school or equivalent/bachelor or equivalent/master or above/unclear), ethnicity (i.e., Han/minority), family socioeconomic status (SES), district (nine districts in total), and school (135 schools in total). Age- and sex-specific BMI, calculated from self-reported height and weight, was used as a covariate to determine weight status based on weight and height, following China’s norm reference data [40], classifying participants into three categories (1) normal weight & underweight, (2) overweight, and (3) obese. SES was measured using the adapted version of the MacArthur Scale of Subjective Social Status (a 10-rung ladder with higher scores indicating better SES) [41]. In the previous studies, those variables were controlled for in the statistical analyses to minimize the confounding bias [23, 24, 26, 27, 42]. Sleep time was measured using the Pittsburgh sleep quality index (PSQI) and the Chinese version of the PSQI has been verified among Chinese adolescents [43]. Screen time was assessed using the relevant items in the Health Behavior of School-aged Children (HBSC) survey, including the time spent on various screen activities (i.e., TV/movies, video games and other screen-based activities during leisure time) in the past seven days. The average daily screen time was calculated by the following formula: ([sum of screen time on weekdays × 5] + [sum of weekend day screen time × 2])/7. PA was assessed by one question adapted from the HBSC survey, that was “How many days did you engage in moderate to vigorous PA at least 60 min on weekdays over the past week? (0 = none, 1 = 1 day, 2 = 2 days, 3 = 3 days, 4 = 4 days, 5 = 5 days, 6 = 6 days, and 7 = 7 days)”. This item has been shown satisfactory reliability among Chinese adolescents [44] and has been used widely in Chinese children and adolescents [32, 34, 45, 46]. Sleep duration, screen time and MVPA was respectively dichotomized into meeting or not meeting the guidelines according to the Canadian 24-Hour movement guidelines for children and youth [47] and has been used in Chinese study [48].

### Statistical analysis

After the exclusion of samples with invalid or incomplete data, the formal analysis encompassed a total of 67,281 participants. The statistical analyses were conducted using STATA BE 18.0 (College Station, Texas, USA). To summarize the characteristics of the sample, descriptive statistics were employed. Given the nested nature of the data across different layers, a three-level mixed multilevel effect model was utilized for analysis (with the district as level 3, school as level 2, and individual as level 1), reflecting the sampling strategy. This model was established for examination of the association between independent and dependent. Considering the nature of dependents as ordinal variables, a series of ordinal models were develop to evaluate these associations while adjusting for sociodemographic (e.g., sex) and lifestyle (e.g., PA) variables. Specifically, models 1 and 2 assessed the association of days of MSE (reference group: 0 days) with depressive (reference group: none) and anxiety symptoms (reference group: none). Then, sex- and grade- split models were established to examine the associations in different subgroups. Results were reported as odds ratios (OR) with 95% confidence intervals (CI), establishing statistical significance at a *p*-value less than 0.05.

## Results

Table 1 shows sample characteristics of this study. The mean age of study participants was 13.3 (± 1.8) years. Sample distribution by sex was balanced, with 51.9% boys (*n* = 34,909) and 48.1% girls (*n* = 32,372). Educational stages were indicated as primary school (41.5%), middle school (40.3%), and high school (18.1%). Weight categories included normal (68.1%), overweight (13.5%), and obese (18.4%, *n* = 12,413). Participants' familial context showed a predominance of non-single child households (74.2%) and dual-parent families (93.4%). Parental education was reported with levels ranging from middle school or below to master’s degree or above. Ethnicity was predominantly in Han (96.6%), with a minority of other ethnicities (3.4%). Days of MSE ranged from 0 day to 7 days per week, with the majority being no more than 3 days per week (24.9% for 0 day; 18.1% for 1 day; 18.4% for 2 days; 15.9% for 3 days). Mental health status, stratified by the presence and severity of depressive and anxiety symptoms, showed a majority without symptoms (56.39% for depressive; 68.4% for anxiety), with a very small proportion of participants at the “severe” level (2.4% for depression; 3.9% for anxiety).

**Table 1:** Descriptive characteristics of participants

Continuous variables		Mean	SD
Age (years)		*13*	1.8
Subjective family socioeconomic status		*5*	1.7
***Categorical variables***		***n***	**%**
Sex	Boys	34,909	51.9
	Girls	32,372	48.1
Grade	Primary school	27,954	41.5
	Middle school	27,124	40.3
	High school	12,203	18.1
Weight status	Normal	45,817	68.1
	Overweight	9,051	13.5
	Obese	12,413	18.4
Siblings	Only child	17,354	25.8
	Non-only child	49,927	74.2
Family structure	Both parents	62,836	93.4
	Single	4,445	6.6
Paternal education	Middle school or below	14,619	21.7
	High school or equivalent	18,159	27
	Bachelor or equivalent	26,030	38.7
	Master or above	2,796	4.2
	Unclear	5,677	8.4
Maternal education	Middle school or below	17,617	26.2
	High school or equivalent	18,706	27.8
	Bachelor or equivalent	23,922	35.6
	Master or above	1,635	2.4
	Unclear	5,401	8
Nationality	Han	65,027	96.6
	Minority	2,254	3.4
Number of physical activity, screen time and sleep guidelines met	0	19,277	28.7
	1	36,712	54.6
	2	10,174	15.1
	3	1,118	1.7
Days of muscle-strengthening exercise	0 days	16,780	24.9
	1 day	12,165	18.1
	2 days	12,395	18.4
	3 days	10,726	15.9
	4 days	4,518	6.7
	5 days	5,338	7.9
	6 days	1,068	1.6
	7 days	4,291	6.4
Depressive symptoms	None	37,940	56.4
	Mild	18,764	27.9
	Moderate	6,189	9.2
	Moderate to severe	2,741	4.1
	Severe	1,647	2.4
Anxiety symptoms	None	46,019	68.4
	Mild	14,827	22
	Moderate	3,840	5.7
	Severe	2,595	3.9

*SD *Standard deviation. Subjective family socioeconomic status was scaled from 0 to 10

Table 2 exhibits the adjusted ORs with 95% CIs for the association of days of MSE with depressive symptoms. The ORs for the overall sample indicated a protective effect of days of MSE against depressive symptoms, with ORs decreasing from 0.83 for 1 day to 0.76 for 6 days, and a slight increase to 0.83 for 7 days. For boys, the ORs ranged from 0.80 for 1 day to 0.70 for 6 days, with a rise to 0.76 for 7 days. For girls, the ORs decreased from 0.86 for 1 day to 0.82 for 6 days, with an increase to 0.97 for 7 days.

**Table 2:** Associations between days of muscle-strengthening exercise and depressive symptoms in overall sample and sample by sex

Days of muscle-strengthening exercise	Overall			Boy			Girl		
	OR	95%CI		OR	95%CI		OR	95%CI	
0 days	REF			REF			REF		
1 day	0.83	0.79	0.87	0.80	0.75	0.86	0.86	0.80	0.91
2 days	0.78	0.75	0.82	0.75	0.70	0.80	0.82	0.77	0.88
3 days	0.77	0.74	0.81	0.75	0.69	0.80	0.80	0.75	0.86
4 days	0.79	0.74	0.85	0.75	0.68	0.82	0.85	0.77	0.93
5 days	0.74	0.70	0.79	0.70	0.64	0.76	0.79	0.72	0.87
6 days	0.76	0.67	0.86	0.70	0.59	0.84	0.82	0.68	1.00
7 days	0.83	0.77	0.89	0.76	0.69	0.84	0.97	0.85	1.10

All models controlled all the sociodemographic variables

*OR *Odds ratio, *CI *Confidence interval, *REF *Reference group

Table 3 shows the adjusted ORs and 95% CIs by grade group. For primary school group, the ORs for depressive symptoms decreased from 0.87 for 1 day to 0.58 for 6 days, and then increased to 0.85 for 7 days. Middle school group exhibited ORs ranging from 0.79 for 1 day to 0.70 for 5 days, with an increase to 0.79 for 7 days. High school groups’ ORs decreased from 0.80 for 1 day to 0.65 for 5 days, with a substantial increase to 1.12 for 6 days.

**Table 3:** Associations between days of muscle-strengthening exercise and depressive symptoms in sample by grade group

Days of muscle-strengthening exercise	Primary school			Middle school			High school		
	OR	95%CI		OR	95%CI		OR	95%CI	
0 days	REF			REF			REF		
1 day	0.87	0.81	0.93	0.79	0.73	0.86	0.80	0.73	0.88
2 days	0.85	0.79	0.92	0.73	0.68	0.80	0.71	0.65	0.78
3 days	0.78	0.72	0.85	0.74	0.68	0.80	0.76	0.67	0.87
4 days	0.80	0.72	0.90	0.76	0.68	0.84	0.85	0.69	1.05
5 days	0.80	0.72	0.90	0.70	0.64	0.77	0.65	0.54	0.78
6 days	0.58	0.46	0.73	0.80	0.68	0.95	1.12	0.72	1.74
7 days	0.85	0.75	0.97	0.79	0.70	0.88	0.83	0.65	1.05

All models controlled all the sociodemographic variables

*OR *Odds ratio, *CI *Confidence interval, *REF *Reference group

In Table 4, the overall sample showed an association pattern where the ORs of anxiety symptoms decreased as days of MSE increased up to 6 days per week, with ORs declining from 0.87 for 1 day to 0.79 for 5 days. However, at 7 days of exercise per week, the OR increased up to 0.96. For boys, the ORs decreased from 0.85 for 1 day to 0.77 for 6 days, with an increase to 0.89 or 7 days. For girls, the ORs showed a decrease from 0.89 for 1 day to 0.80 for 5 days, with a notable increase to 1.13 for 7 days.

**Table 4:** Associations between days of muscle-strengthening exercise and anxiety symptoms in overall sample and sample by sex

Days of muscle-strengthening exercise	Overall			Boy			Girl		
	OR	95%CI		OR	95%CI		OR	95%CI	
0 days	REF			REF			REF		
1 day	0.87	0.83	0.92	0.85	0.78	0.91	0.89	0.83	0.96
2 days	0.83	0.79	0.87	0.80	0.74	0.86	0.85	0.79	0.91
3 days	0.85	0.81	0.90	0.84	0.77	0.91	0.87	0.80	0.94
4 days	0.90	0.84	0.97	0.87	0.78	0.96	0.94	0.84	1.04
5 days	0.79	0.74	0.85	0.77	0.70	0.85	0.80	0.73	0.89
6 days	0.87	0.76	1.00	0.77	0.64	0.94	0.99	0.81	1.21
7 days	0.96	0.89	1.05	0.89	0.80	0.99	1.13	0.98	1.29

All models controlled all the sociodemographic variables

*OR *Odds ratio, *CI *Confidence interval, *REF *Reference group

Table 5 demonstrates the association between days of MSE and anxiety symptoms across different grade groups. For primary school group, the ORs decreased from 0.95 for 1 day to 0.87 for 6 days, with an increase to 1.01 for 7 days. Middle school group showed ORs decreasing from 0.81 for 1 day to 0.82 for 6 days, with an increase to 0.92 for 7 days. High school group's ORs decreased from 0.84 for 1 day to 0.65 for 5 days, with a subsequent increase to 0.97 for 7 days.

**Table 5:** Associations between days of muscle-strengthening exercise and anxiety symptoms in sample by grade group

Days of muscle-strengthening exercise	Primary school			Middle school			High school		
	OR	95%CI		OR	95%CI		OR	95%CI	
0 days	REF			REF			REF		
1 day	0.95	0.87	1.03	0.81	0.74	0.89	0.84	0.77	0.93
2 days	0.91	0.84	0.99	0.79	0.72	0.86	0.73	0.66	0.81
3 days	0.88	0.80	0.97	0.82	0.76	0.90	0.80	0.69	0.92
4 days	0.93	0.82	1.05	0.89	0.80	1.00	0.76	0.60	0.95
5 days	0.84	0.74	0.96	0.77	0.70	0.85	0.65	0.53	0.80
6 days	0.87	0.68	1.11	0.82	0.68	0.99	1.13	0.71	1.79
7 days	1.01	0.88	1.16	0.92	0.82	1.04	0.97	0.75	1.24

All models controlled all the sociodemographic variables

*OR *Odds ratio, *CI *Confidence interval, *REF *Reference group

## Discussion

This study explored the associations of days of MSE with depressive and anxiety symptoms, respectively, using a large sample of Chinese children and adolescents. This study has the following findings: (1) more days of MSE were associated with lower likelihoods for depressive and anxiety symptoms, respectively; (2) the associations of days of MSE with depressive and anxiety symptoms were not consistent across different subgroups (i.e., sex and grade).

Results of the current study found that more days participating in MSE, for children and adolescents, were negatively associated with lower likelihood of depressive symptoms. Synthesized evidence has demonstrated the effects of MSE on treating depressive symptoms. One recent study using the Network Meta-analysis indicates that strength training is one of the effective approaches against depressive symptoms (Hedge’s g −0.49, 95%CI: −0.69 to −0.29) [30]. Our finding is also consistent with a few studies conducted in children and adolescents. For example, a cross-sectional study found that more MSE participations in children and adolescents were associated with lower odds of depressive symptoms. Another intervention study (randomised control trial) revealed that MSE could lead to reductions in depressive symptoms in adolescents with obesity. One study conducted by Burns et al. suggested that adhering to MSE guidelines was associated with lower prevalence of feeling sad or hopeless (proxy measure of depressive symptoms). These findings are parallelled with the current study, providing supporting evidence on the association between more MSE participations and lower likelihood of depressive symptoms. On the contrary, the association between MSE and depressive symptoms observed in this study contradicts with one study by Shi et al. [50]. One possible reason is that their study used proxy measure of depressive symptoms, whereas our study used an established scale. This discrepancy implies that future studies should use more standardized instruments when exploring the association between MSE and health indicators in children and adolescents. In adult population, a large number of studies have examined the association between MSE and depressive symptoms, which could in part support the findings observed in the current study. For example, using the German nationally representative data, Bennie et al., found that MSE was associated with reduced prevalence ratios for all levels of depressive symptom severity compared to those who did not participate in MSE. Such a finding was also found in the US and UK samples. 

In this study, a negative association between days of MSE and anxiety symptoms in children and adolescents was found. This association is firstly observed in children and adolescents in the literature, to our knowledge, which accordingly has no direct evidence for comparison. Nevertheless, the association between days of MSE and anxiety symptoms in adults has been observed [28, 31, 51], and these studies can in part support our study. For example, a randomized controlled trial study found that muscle-strengthening training can significantly reduce anxiety symptoms from baseline to post-intervention [31]. The findings from O’Connor et al. indicated that MSE was associated with reductions in anxiety symptoms among healthy adults [51]. Another cross-sectional study using a large sample of UK adults revealed the negative association of higher frequency of MSE with lower anxiety symptoms [28]. There are also some other studies that can support the association between MSE participations and anxiety symptoms in adults [29]. Therefore, these findings highlight the potential of MSE in reducing anxiety symptoms, even in adults. Given the absence of direct evidence among children and adolescents, our findings underscore the need for further research to confirm this association. Anxiety symptoms decrease as the frequency of MSE increases. However, a slight increase in anxiety is observed when exercising every day. These findings suggest that incorporating MSE into weekly routines can be an effective strategy for managing anxiety. However, it is important to balance exercise with rest to avoid potential increases in anxiety from over-exercising.

In this study, some counterintuitive findings related to sex and age-related variations in the associations of days of MSE with depressive and anxiety symptoms have to be mentioned. This finding, to the best of our knowledge, is relatively novel to the research area as very few studies examined the sex and age-related differences. For example, in some studies conducted in adult populations, they did not find sex and age-related differences in the associations of days of MSE with depressive and anxiety symptoms [23, 24, 26, 27]. This lack of evidence suggests that future studies should focus more on the potential sex and age-related differences. In this study, we surprisingly observed that in girls, participating in some days (e.g., 6 or 7 days) of MSE was not significantly associated with depressive and anxiety symptoms in comparison to those without MSE. Similar results were also reflected in high school students for depressive symptoms and primary school students for anxiety symptoms. These findings indicated that the pattern of associations of days of MSE with depressive and anxiety symptoms could be inconsistent across different subgroups. Sex differences in the associations between days of MSE and depressive/anxiety symptoms are primarily driven by several biological mechanisms, including hormonal fluctuations and neurobiological processes [52, 53]. These differences should be explained by subgroup sample characteristics, such as distribution of depressive symptoms, anxiety and stress responses [54]. Although explanations to the differences would beyond our study aim, future studies are encouraged to address. This exploration would be beneficial to develop sex- and age-specific mental health interventions.

It is likely that there is no single mechanism that can explain the association between MSE and mental health disorder, but mechanisms at behavioural, psychological and biological level may be useful. From the behavioural perspective, there is evidence to demonstrate a positive association between MSE and higher levels of PA [35], which in turn could reduce the risks of depressive [11, 12, 15] and anxiety symptoms [13, 15, 16]. Psychologically, more participations of MSE may result in positive mental wellness against negative mental health disorder (e.g., depressive symptoms). For example, one study indicated that higher frequency of MSE were associated with increased levels of subjective wellbeing and resilience [25], which in turn lowers the development of depressive and anxiety symptoms. Similarly, MSE is associated with higher levels of self-esteem, self-confidence, and a positive body image [22, 55, 56]. These psychological attributes are important in mitigating the risk of depressive and anxiety symptoms. Furthermore, biological related mechanisms should be mentioned. One systematic review and meta-analysis suggests that higher muscular strength is associated with lower risks for depressive symptoms [57]. Enhancing muscle strength may decrease depressive and anxiety symptoms through a combination of biological processes including increased neuroplasticity, neurophysiological adaptations, hormonal adjustments, and inflammation reduction [51]. However, it is challenging to know whether the mechanism linking MSE and mental health disorder works in isolation or in combination. Accordingly, future studies are needed to address the uncertainty.

### Implication for practice and research

This is one of the very few studies assessing the associations of MSE days with mental health disorder outcomes (i.e., depressive and anxiety symptoms) in children and adolescents and extends the scientific evidence on the mental health benefits of MSE participations, especially in Chinese young population. It is beneficial to provide insights into for the relevant Chinese stakeholders (e.g., educational policymakers, schoolteachers, family guardians) to encourage children and adolescents to do more muscle-promoting activities for mental health promotion. For school-based physical activity related intervention, it is acceptable to incorporate MSE as one of the intervention approaches or components [58]. However, we have to admit that studies on the association between days of MSE and depression and anxiety symptoms in children and adolescents are relatively fewer and receive less research attention compared with those in adults. This limitation hinders our ability to directly compare our study with the existing body of evidence. Further research in this area is crucial for building a more robust evidence base.

### Study strengths and limitations

This study certainly has some strengths. The first one is the inclusion of a series of covariates controlled in statistical analysis, thereby increasing the robustness of results. Nevertheless, some study limitations have to be mentioned. The study sample was from Shenzhen, China, and may not fully represent children and adolescents nationwide due to geographical, economic, and cultural differences. Owing to the cross-sectional study design, it is not possible to infer causal associations. More improved studies are needed to reveal the casual association between 24-hour movement guidelines and academic performance in children and adolescents and future studies should seek to elucidate the underlying mechanism(s) linking the 24-hour movement guidelines and academic performance. Self-reported questionnaires were used to collect data on all the variables, which is subject to study participants’ recall and social desirability biases. Therefore, some measurement issues (e.g., not capturing all kinds of recreational screen time) should be considered when interpreting study findings. Although we used a validated measure, a single question may not fully capture the nuances of MSE across different age groups. Future studies should consider more comprehensive assessments. Additionally, while overweight and obesity can increase the risk of depressive and anxiety symptoms, our study only adjusted for BMI as a covariate. Furthermore, some important confounding factors were not included in the present study.

## Conclusion

Findings of this study indicated the negative association between MSE and depressive and anxiety symptoms in children and adolescents. The negative association persists across various subgroups, underscoring the value of interventions focused on enhancing participations of MSE, as a standalone approach . Given the rising mental health problems in children and adolescents, such findings highlight the critical role of accessible prevention and intervention strategies tailored to improving mental health. Owing to some inherent study limitations, future studies are required to verify the associations observed in this study.

## Data Availability

The data analyzed in this study are available upon request from the corresponding author.
